# Relationship between muscle strength and dyslipidemia, serum 25(OH)D, and weight status among diverse schoolchildren: a cross-sectional analysis

**DOI:** 10.1186/s12887-018-0998-x

**Published:** 2018-02-02

**Authors:** Caitlin E. Blakeley, Maria I. Van Rompay, Nicole S. Schultz, Jennifer M. Sacheck

**Affiliations:** Friedman School of Nutrition Science and Policy, Tufts University, 150 Harrison Avenue, Boston, MA 02111 USA

**Keywords:** Grip strength, BMI z-score, 25(OH)D, Blood lipids, Cardiometabolic risk factors

## Abstract

**Background:**

The relationship between muscle strength and cardiometabolic risk factors in youth, and the potential influence of vitamin D status on this relationship, is not well understood. This study examined associations between muscle strength and dyslipidemia, serum 25-hydroxyvitamin D [25(OH)D], and weight status in diverse schoolchildren.

**Methods:**

Measures of hand-grip strength (standardized for sex and body weight), anthropometrics (height and weight converted to BMI z-score [BMIz]), sociodemographics, and fasting blood concentrations of plasma HDL-C and triglycerides and serum 25(OH)D were collected from 350 4th-8th grade schoolchildren (11.2 ± 1.3 y, 49.4% female, 56.3% non-white/Caucasian). Logistic regression was used to measure associations between standardized tertiles of grip strength and blood lipids, 25(OH)D, and weight status along with associations between 25(OH)D and dyslipidemia and weight status.

**Results:**

Children with higher grip strength had lower odds of overweight/obesity (OR: 0.03, 95% CI: 0.01-0.06, in the highest tertile of grip strength vs. lowest, *p* for trend< 0.0001), borderline/low HDL-C (OR: 0.28, 95% CI: 0.16-0.50, *p* for trend< 0.0001), and borderline/high triglycerides (OR: 0.48, 95% CI: 0.25-0.92, *p* for trend< 0.05), adjusting for covariates. Associations between blood lipids and grip strength became non-significant after further adjustment for BMIz. No association was observed between grip strength and 25(OH)D, nor between 25(OH)D and borderline/low HDL-C or weight status; however, vitamin D sufficiency was associated with lower odds of borderline/high triglycerides compared with vitamin D deficiency (OR: 0.26, 95% CI: 0.09-0.74, p for trend< 0.05) before BMIz adjustment.

**Conclusion:**

Among racially/ethnically diverse children, muscle strength was associated with lower dyslipidemia. Longitudinal studies are needed to explore whether changes in muscle strength impact this relationship in children, independent of weight status.

**Trial registration:**

This study was registered at www.clinicaltrials.gov (No. NCT01537809) on February 17, 2012.

## Background

In both children and adolescents, studies have demonstrated adverse effects of low cardiorespiratory fitness on individual and clustered cardiometabolic risk factors, including body mass index (BMI), triglycerides, HDL-cholesterol (HDL-C), LDL-cholesterol (LDL-C), and blood pressure [1–6]. These studies determined that higher cardiorespiratory fitness in children is associated with healthier lipid profiles and a reduced occurrence of cardiometabolic risk factors in adulthood. Another key component of fitness is muscular strength, although its links to cardiometabolic risk are less well studied. While a growing body of evidence has demonstrated protective effects of muscle strength on cardiometabolic risk factors in adults [7, 8], fewer studies have examined this relationship in children and adolescents [1, 9, 10].

One prospective cohort study suggested that greater isometric back and abdominal strength in Danish youth is associated with lower levels of cardiometabolic risk factors in young adulthood independent of cardiovascular fitness and adiposity [10]. A systematic review concluded that muscle strength improvements between childhood and adolescence are inversely associated with overall changes in adiposity. However, evidence for the association between changes in muscle strength and other cardiometabolic risk factors was inconclusive given the limited number of studies [6]. Furthermore, the Institute of Medicine’s (IOM) 2012 Fitness Measures and Health Outcomes in Youth report highlighted the dearth of literature examining the association between musculoskeletal fitness and health outcomes in youth, independent of potential modifiers [9]. As such, the report called for robust analyses of this relationship and recommended grip strength as a valid measure of musculoskeletal fitness in youth. While evidence is emerging for an association between muscle strength and various health outcomes in children and adolescents [1, 10], additional research is warranted to determine whether an association between grip strength and cardiometabolic risk factors in these age groups exists.

A separate body of literature has demonstrated a positive relationship between muscular strength and vitamin D status (assessed using serum 25-hydroxyvitamin D [25(OH)D]) in adults, [11, 12] but this relationship is inconsistent in studies of children and youth [13, 14]. Vitamin D adequacy has also shown beneficial relationships with several cardiometabolic outcomes, including blood pressure, serum lipids, and insulin and glucose metabolism. Evidence for a direct cause-and-effect relationship between vitamin D status and cardiometabolic risk factors in youth is still under investigation [15]; however some proposed mechanisms include the presence of vitamin D receptors on pancreatic β cells, as well as cells of the blood vessel wall. By binding to its receptors, 1,25 dihydroxyvitamin D may confer vasculoprotection, decreased insulin resistance, as well as anti-inflammatory effects [16, 17]. Given the potential positive impact of vitamin D status on both muscle strength and cardiometabolic risk, it is important to consider whether vitamin D may play a role in the relationship between muscle strength and cardiometabolic risk.

The primary aim of the present study was to examine associations between muscle strength and dyslipidemia (HDL-C, triglycerides) in addition to weight status among a diverse sample of urban schoolchildren. Furthermore, as vitamin D has known relationships with both muscle strength [18] and cardiometabolic risk [19] in adults, we investigated whether serum 25(OH)D may modulate the strength/cardiometabolic risk relationship by examining associations between vitamin D status and both muscle strength and dyslipidemia.

## Methods

### Study design and study sample

The study sample utilized for this analysis was a sub-sample of children participating in the Daily D Health Study (DDHS), which was a randomized, double-blind trial that assessed the impact of 6 months of daily vitamin D_3_ supplementation (600 IU, 1000 IU, or 2000 IU) on serum 25(OH)D and cardiometabolic risk factors in a multi-ethnic sample of schoolchildren in the fourth through eighth grades during the 2011-2012 and 2012-2013 school years. Children were recruited from public elementary and middle schools in four urban school districts in the greater Boston, MA area. Detailed descriptions of the DDHS study protocol and recruitment have been published elsewhere [20].

Grip strength measures were collected during the 2012-2013 school year at the baseline study visit (prior to vitamin D supplementation) on 381 children. Children diagnosed with diabetes, missing grip strength data, or who were underweight at baseline were excluded (*N* = 31), leaving 350 children (11.2 ± 1.3 y; 49.4% female) in the analytic sample. All study visits were conducted in person at the school of enrollment. The protocol was reviewed and approved by the Tufts University Institutional Review Board. Both written parental informed consent and the child’s written assent were obtained before inclusion in the study.

### Grip strength

Grip strength was measured using a digital handgrip dynamometer (T.K.K.5401, Takei Scientific Instruments Co., Ltd., Niigata, Japan). The machine was adjusted to an appropriate setting to ensure that the second joint of the index finger was at a 90-degree angle on the handle (90° flexion between proximal and middle phalangeal joint). Children were instructed to stand with feet hip-width distance apart, not to hold their breath, and to squeeze for 10-15 s, or until the force generated plateaued. Research assistants conducted a practice test on each subject’s dominant hand prior to the actual test. Two measures were then conducted on each hand, either switching hands in cases where grip size was the same on each hand, or with a 60 s rest interval between measures when it was not. The average grip strength was calculated from all four trials. Grip strength was subsequently expressed per kilogram of body weight to account for differences in body size. Based on prior literature, grip strength was further standardized by sex and age [21, 22], creating a z-score to remove the effect of age and sex differences. The z-score was calculated by first subtracting the study population’s mean grip strength per kilogram of body weight from each subject’s grip strength per kilogram of body weight. This value was subsequently divided by the grip strength standard deviation for each sex and age group. Lastly, the z-score was categorized into tertiles of low (<− 0.45 kg), moderate (− 0.45 to 0.33 kg), and high grip strength (> 0.33 kg) [10].

### Blood measures

Blood was drawn from the antecubital vein on the study morning following an overnight fast for measurement of plasma HDL-C and triglycerides, and serum 25(OH)D. Concentrations of HDL-C and triglycerides were measured with the Hitachi 917 analyzer using reagents and calibrators from Roche Diagnostics (Indianapolis, IN) in a laboratory certified by the Centers for Disease Control and Prevention (CDC)/National Heart, Lung, and Blood Institute Lipid Standardized Program. HDL-C and triglyceride concentrations were categorized according to the National Cholesterol Education Program (NCEP) cut-points [23]; HDL-C was classified as borderline/low (≤45 mg/dL) or normal (> 45 mg/dL). Triglycerides were standardized by age and classified as borderline/high (≥75 mg/dL for children ≤9 years; ≥90 mg/dL for children > 9 years) or normal (< 75 mg/dL for children ≤9 years; < 90 mg/dL for children > 9 years).

Total serum 25(OH)D was measured using the validated liquid chromatography-mass spectrometry (LC-MS/MS) method including fractionation of 25(OH)D_3_ and 25(OH)D_2_ in serum [24]. 25(OH)D samples from study subjects were prepared and analyzed through a turbulent flow LC system (Cohesive Technologies, Franklin, MA) followed by traditional laminar flow chromatography. The study samples were then analyzed relative to the control solutions (NIST vitamin D standard references) for detection and quantification of the 25(OH)D_3_ and 25(OH)D_2_ component of each sample. The analysis was performed using a TSQ Quantum Ultra triple mass-spectrometer (Thermo Finnigan Corp., San Jose, CA). The intra-assay coefficient of variation is 6.0%. Serum 25(OH)D status was classified as deficient (< 20 ng/ml), insufficient (≥20 to < 30 ng/ml), or sufficient (≥ 30 ng/mL) according to IOM criteria [25].

### Sociodemographic measures

Age was determined from the parent-reported birth date. Race/ethnicity was reported by parent questionnaire as white/Caucasian, black/African American, Mexican/Mexican American, other Hispanic/Latino, Asian/Asian American, Native American, multi-racial, or other race/ethnicity. For these analyses, race/ethnicity was consolidated into white/Caucasian, black/African American, Hispanic/Latino, Asian, or multiracial/other categories. Parents also reported whether their child was eligible for free or reduced-price school meals, as a proxy measure of socioeconomic status. Sedentary time was ascertained using the Block Kids Physical Activity Screener (NutritionQuest, Berkeley, CA) and was calculated based on the number of hours per day spent watching television or videos, or using a computer.

### Anthropometric measures and pubertal status

Height and weight were measured in triplicate with light clothing and no shoes. Height was measured using a portable stadiometer (Model 214, Seca Weighing and Measuring Systems, Hanover, MD), and weight was measured using a digital platform scale (Model 803, Seca Weighing and Measuring Systems, Hanover, MD). BMI z-score (BMIz) was calculated using the CDC sex-specific growth charts. Weight status was classified into two groups: healthy weight (BMI < 85th percentile for age) and overweight/obese (BMI ≥ 85th percentile for age) based on the CDC cut-points.

Pubertal status was classified into two groups: pre-puberty/early puberty or late puberty/post-puberty. Using a brief, validated questionnaire [26], female subjects were asked if they had reached menarche (yes/no) and male subjects were asked if their voice had changed (not yet started/barely started/definitely underway/seems complete). If a girl answered, “yes” for menarche or a boy answered “definitely underway” or “seems complete” for voice change, then the child was categorized as late pubertal/post-pubertal.

### Statistical analyses

Pearson’s Chi-square test was used to determine the distribution of children for each categorical variable across tertiles of grip strength. Continuous variables were compared by grip strength tertiles using ANOVA if the covariate was normally distributed; otherwise, the Kruskal Wallis test was applied. Covariates were selected based on prior literature investigating grip strength and cardiometabolic risk factors [10, 27], and included age, sex, pubertal status, sedentary time, free/reduced price lunch, race/ethnicity, and BMIz.

To examine whether there were any significant associations between tertiles of grip strength and the four outcomes of interest (HDL-C, triglycerides, BMIz, and vitamin D status), four models were built for each outcome variable, with each model adjusted for a specific set of covariates. The first model was adjusted for age and sex. Model two was additionally adjusted for pubertal status, sedentary time, free/reduced price lunch, and race/ethnicity. The third model for each outcome was further adjusted for BMIz, [10] except when obesity was the outcome of interest. Tolerance tests were performed to assess collinearity of variables within the second and third models. In a fourth model, vitamin D status was added to the covariates in model two to further examine whether vitamin D status modulates the relationship between grip strength and cardiometabolic risk [27]. For each model, a test for linear trend across tertiles of grip strength was performed by assigning children within each tertile the median value of grip strength for that tertile and including these values as a continuous variable in regression models [28].

Similarly, to examine associations between vitamin D status and borderline/low HDL-C, borderline/high triglycerides, and BMIz, three separate logistic regression models were constructed for each outcome. Each model was adjusted for the aforementioned covariates. All analyses were performed using SAS statistical software (version 9.3; SAS Institute, Cary, NC), and values of *p* < 0.05 were considered statistically significant.

## Results

In the overall sample of 350 schoolchildren (49.4% female, 11.2 ± 1.3 y, 56.3% non-white/Caucasian), 48.6% of children were overweight/obese, 66.9% were eligible for free/reduced price lunch, and 58.6% reported being sedentary for ≥2 h/day. Serum vitamin D status was classified as sufficient in 11.4% (*n* = 40) of schoolchildren, while 52.6% (*n* = 184) were vitamin D insufficient and 36% (*n* = 126) were vitamin D deficient. Plasma HDL-C was considered borderline/low in 41.1% (*n* = 144) of schoolchildren, while triglycerides were borderline/high in 25.1% (*n* = 88).

Additional sociodemographic and other characteristics are shown by tertile of grip strength in Table 1. Grip strength was inversely associated with weight, height, and BMIz (*p* < 0.0001). Among those with high grip strength, more children were normal weight (82.1%) than overweight/obese (18%); the inverse weight status distribution was observed in the low grip strength tertile. Children with higher grip strength demonstrated higher HDL-C (*p* = 0.0001) and a trend toward lower triglycerides (*p* = 0.07). Other characteristics such as sex, age, race/ethnicity, pubertal status, sedentary time, and free/reduced price lunch did not differ by grip strength tertile (*p* > 0.05).

**Table 1:** Sociodemographic, behavioral, and health status characteristics by tertile of grip strength in schoolchildren aged 9-14 (*N* = 350)

	Tertiles of grip strength			
	Low (*N* = 116)	Moderate (*N* = 117)	High (N = 117)	*p* value
Grip strength (kg)^a^	−0.95 (− 1.32, − 0.66)	−0.02 (− 0.23, 0.15)	1.04 (0.67, 1.36)	
Sex				
Male	60 (51.7)	61 (52.1)	56 (47.9)	0.77
Female	56 (48.3)	56 (47.9)	61 (52.1)	
Age (y)	11.4 ± 1.3	11.1 ± 1.3	11.2 ± 1.3	0.12
Race/ethnicity				
White/Caucasian	48 (41.4)	51 (43.6)	54 (46.2)	0.64
Black/African American	13 (11.2)	15 (12.8)	22 (18.8)	
Hispanic/Latino	27 (23.3)	28 (23.9)	19 (16.2)	
Asian	7 (6.0)	7 (6.0)	6 (5.1)	
Multiracial/Other	21 (18.1)	16 (13.7)	16 (13.7)	
Pubertal status^b^				
Pre-puberty/early puberty	80 (69.0)	78 (66.7)	75 (64.1)	0.73
Late puberty/post-puberty	36 (31.0)	39 (33.3)	42 (35.9)	
Free/reduced price lunch				
Yes	75 (64.7)	84 (71.8)	75 (64.1)	0.38
No	41 (35.3)	33 (28.2)	42 (35.9)	
Sedentary time^c^				
< 2 h/day	43 (37.4)	48 (41.0)	53 (45.7)	0.44
≥ 2 h/day	72 (62.6)	69 (59.0)	63 (54.3)	
Weight (kg)	63.3 ± 18.4	47.0 ± 11.0	42.8 ± 10.2	< 0.0001
Height (cm)	155 ± 11.7	149 ± 9.4	150 ± 11.2	< 0.0001
BMIz ^d^	1.7 (1.3, 2.2)	0.9 (0.4, 1.4)	0.2 (−0.3, 0.8)	< 0.0001
Weight Status				
Normal weight	17 (14.7)	67 (57.3)	96 (82.1)	< 0.0001
Overweight/obese	99 (85.3)	50 (42.7)	21 (18.0)	
HDL-C (mg/dL)	45.3 ± 11.4	50.9 ± 12.6	50.7 ± 9.6	0.0001
HDL-C status^e^				
Borderline/low	65 (56.0)	49 (41.9)	30 (25.6)	< 0.0001
Normal	51 (44.0)	68 (58.1)	87 (74.4)	
Triglycerides (mg/dL)^d^	69.0 (52.0, 96.5)	63.0 (47.0, 90.0)	62.0 (49.0, 77.0)	0.07
Triglyceride status^f^				
Normal	80 (69.0)	86 (73.5)	96 (82.1)	0.06
Borderline/high	36 (31.0)	31 (26.5)	21 (18.0)	
Serum 25(OH)D (ng/mL)	21.9 ± 6.8	22.1 ± 6.1	22.6 ± 6.7	0.71
Vitamin D status^g^				
Deficient	43 (37.1)	42 (35.9)	41 (35.0)	0.96
Insufficient	58 (50.0)	63 (53.9)	63 (53.9)	
Sufficient	15 (12.9)	12 (10.3)	13 (11.1)	

Differences between tertiles of grip strength were determined using chi-square test and ANOVA. Data are mean ± standard deviation or n (%), unless otherwise stated; level of significance was *p* < 0.05

*Abbreviations*: *BMIz* BMI z-score, *HDL-C* HDL-cholesterol, *25(OH)D* 25-hydroxyvitamin D

^a^Tertiles of grip strength are standardized for age, sex, and body weight and are the median (IQR)

^b^Late puberty/post-puberty defined as reported voice change (male) or menarche (female)

^c^Sedentary time defined as the number of hours per day spent watching television, videos, and using a computer

^d^Differences between tertiles of grip strength were determined using the Kruskal Wallis test and data are the median (IQR)

^e^Borderline/Low defined as ≤45 mg/dL; normal defined as > 45 mg/dL

^f^Normal defined as < 75 mg/dL for children ≤9 years and < 90 mg/dL for children older than 9 years; borderline/high defined as ≥75 mg/dL for children ≤9 years and ≥90 mg/dL for children older than 9 years

^g^Deficient defined as < 20 ng/mL; insufficient defined as ≥20 and < 30 ng/mL; sufficient defined as ≥30 ng/mL

Table 2 shows the odds ratios for borderline/low HDL-C, borderline/high triglycerides, overweight/obesity, and vitamin D deficiency for those with a moderate or high grip strength compared to those with a low grip strength. A significant trend across tertiles of grip strength was found for the three cardiometabolic risk factors, with higher grip strength associated with lower odds of borderline/low HDL-C (72% lower, *p* for trend< 0.0001), borderline/high triglycerides (52% lower, *p* for trend = 0.03), and overweight/obesity (100% lower, *p* for trend< 0.0001) after adjustment for covariates except BMIz. After additional adjustment for BMIz, grip strength was no longer associated with odds of borderline/low HDL-C or borderline/high triglycerides (*p* for trend > 0.05). The addition of vitamin D as a covariate did not attenuate associations between grip strength and each cardiometabolic risk factor. No significant association was observed between higher grip strength and vitamin D deficiency after basic and multivariable adjustment; however, when BMIz was added as a covariate, there was a trend toward a direct relationship (*p* for trend = 0.06).

**Table 2:** Odds ratios for cardiometabolic risk factors and vitamin D deficiency with a moderate or high grip strength relative to a low grip strength (*N* = 350)

	Low	Moderate	High	*p* value for trend
	OR (95% CI)	OR (95% CI)	OR (95% CI)	
Borderline/Low HDL-C (*n* = 144)				
Model 1	1.00	0.58 (0.35, 0.98)	0.28 (0.16, 0.48)	< 0.0001
Model 2	1.00	0.57 (0.34, 0.98)	0.28 (0.16, 0.50)	< 0.0001
Model 3	1.00	1.19 (0.64, 2.22)	0.98 (0.47, 2.04)	0.93
Model 4	1.00	0.57 (0.33, 0.97)	0.28 (0.16, 0.49)	< 0.0001
Borderline/High Triglycerides (*n* = 88)				
Model 1	1.00	0.79 (0.44, 1.40)	0.47 (0.25, 0.88)	0.02
Model 2	1.00	0.80 (0.44, 1.46)	0.48 (0.25, 0.92)	0.03
Model 3	1.00	1.36 (0.69, 2.68)	1.24 (0.54, 2.82)	0.62
Model 4	1.00	0.75 (0.41, 1.39)	0.44 (0.23, 0.86)	0.02
Overweight/Obese (*n* = 170)				
Model 1	1.00	0.12 (0.06, 0.22)	0.04 (0.02, 0.07)	< 0.0001
Model 2	1.00	0.11 (0.06, 0.21)	0.03 (0.01, 0.06)	< 0.0001
Model 3	–	–	–	–
Model 4	1.00	0.10 (0.05 0.20)	0.03 (0.01, 0.06)	< 0.0001
Vitamin D Deficient (*n* = 126)				
Model 1	1.00	1.03 (0.62, 1.70)	0.95 (0.57, 1.56)	0.82
Model 2	1.00	1.01 (0.60, 1.70)	0.90 (0.53, 1.52)	0.68
Model 3	1.00	1.60 (0.90, 2.85)	1.90 (0.99, 3.65)	0.06
Model 4	–	–	–	–

Data are odds ratios from logistic regression models. Tertiles of grip strength are standardized for age, sex, and body weight. *N* represents the number of participants with that risk factor

*Abbreviation*: *HDL-C* HDL-cholesterol

Model 1 was adjusted for age and sex

Model 2 was additionally adjusted for pubertal status, sedentary time, free/reduced-price lunch, and race/ethnicity

Model 3 was further adjusted for BMIz

Model 4 was adjusted for the covariates in model 2 as well as for vitamin D status. BMIz was not included in the model

Table 3 presents the odds ratios for borderline/low HDL-C, borderline/high triglycerides, and overweight/obesity for those with vitamin D sufficiency and insufficiency compared to those with vitamin D deficiency. No significant associations were observed between vitamin D status and borderline/low HDL-C nor between vitamin D status and overweight/obesity (*p* > 0.05). A significant trend across tertiles of vitamin D status was found for triglycerides, with vitamin D sufficiency associated with lower odds of borderline/high triglycerides (74% lower, *p* for trend = 0.046). After additional adjustment for BMIz, vitamin D status no longer showed a significant trend with odds of borderline/high triglycerides (*p* for trend > 0.05).

**Table 3:** Odds ratios for cardiometabolic risk factors by vitamin D status (*N* = 350)

	Deficient	Insufficient	Sufficient	*p* value for trend
	OR (95% CI)	OR (95% CI)	OR (95% CI)	
Borderline/Low HDL-C (*n* = 144)				
Model 1	1.00	0.78 (0.49, 1.24)	0.79 (0.38, 1.64)	0.34
Model 2	1.00	0.66 (0.40, 1.09)	0.61 (0.28, 1.33)	0.25
Model 3	1.00	0.77 (0.45, 1.33)	0.93 (0.40, 2.17)	0.88
Borderline/High Triglycerides (*n* = 88)				
Model 1	1.00	0.89 (0.53, 1.49)	0.38 (0.14, 1.06)	0.11
Model 2	1.00	0.66 (0.38, 1.16)	0.26 (0.09, 0.74)	0.046
Model 3	1.00	0.73 (0.40, 1.30)	0.31 (0.10, 0.93)	0.13
Overweight/obese (*n* = 170)				
Model 1	1.00	0.68 (0.43, 1.09)	0.55 (0.27, 1.15)	0.06
Model 2	1.00	0.67 (0.41, 1.09)	0.53 (0.25, 1.15)	0.08
Model 3	–	–	–	

Data are odds ratios from logistic regression models. *N* represents the number of participants with that risk factor

*Abbreviation*: *HDL-C* HDL-cholesterol

Model 1 was adjusted for age and sex

Model 2 was additionally adjusted for pubertal status, sedentary time, free/reduced-price lunch, and race/ethnicity

Model 3 was further adjusted for BMIz

## Discussion

These findings suggest that improved muscle strength may confer cardiometabolic risk benefits, including lower triglycerides and BMIz, as well as higher HDL-C. While longitudinal analyses are necessary to determine causality, these findings suggest muscle strength may be important for improved cardiometabolic health and should be considered in the development of youth physical activity programs and recommendations. Given that dyslipidemia and weight status in youth strongly predicts cardiometabolic health in adulthood [29, 30], improved muscle strength during childhood may be important for the reduction of cardiometabolic risk factors later in life.

Consistent with prior studies [1, 10, 31], we found that children with higher grip strength were at lower risk for poor cardiometabolic health, as measured by borderline/low HDL-C, borderline/high triglycerides, and overweight/obesity. Similarly, Artero and colleagues [1] computed a muscular strength score from handgrip strength and the standing long jump, and found an inverse association between muscular fitness and clustered metabolic risk. In the present study, the significant relationship between grip strength and both HDL-C and triglycerides was eliminated after adjustment for BMIz. This attenuation by weight status is consistent with a previous study [31] that used the sum of voluntary contractile force at four sites (hand-grip, shoulder (extension and flexion), and leg) to determine the association between muscular strength and a clustered cardiovascular risk factor score. This is not surprising given that the negative impact of overweight and obesity on cardiometabolic health in both children and adults is well established [29, 32]. Further research is needed, however, to examine the physiological pathway by which weight status influences the impact of muscle strength on HDL-C and triglycerides. Improved grip strength may result from greater muscle mass or enhanced muscular health and performance, both of which could confer protective effects on lipid metabolism and be reduced in overweight and obese children. Longitudinal studies are therefore also necessary to further elucidate and clarify potential causal pathways.

Our finding of an inverse relationship between grip strength and BMIz is noteworthy. Typically, increased body size confers greater absolute strength [9, 33], which can be explained by adaptive increases in muscle mass to support excess body weight. Greater muscle mass, however, may not equate to improved muscular health or efficiency, which could be implicated in the relationship between muscle strength and cardiometabolic health outcomes. In the present study, when grip strength was not standardized to body weight, the expected positive relationship between BMIz and grip strength was observed (data not shown). However, when grip strength was expressed per kilogram of body weight, the relationship notably changed, and children with lower BMIz demonstrated improved grip strength, suggesting that leaner children may be more muscularly fit. While the odds ratio for overweight/obesity was unusually strong, which could be explained by the standardization of both BMIz and grip strength to body weight, the association should not be discounted and further research should express muscle strength measures in both absolute terms and relative to body weight.

Despite the significant findings for grip strength and cardiometabolic risk factors, we did not observe a significant association between grip strength and vitamin D status. This is surprising given that vitamin D deficiency has been associated with muscle myopathy and weakness, and vitamin D receptor activation by vitamin D has been shown to increase muscle protein synthesis [11]. Possible explanations may be that our study did not include enough children that were vitamin D sufficient (> 30 ng/ml) in this baseline analysis to identify these relationships and that a longitudinal vitamin D supplementation study is warranted. Research examining the association between grip strength and vitamin D in youth is limited and inconsistent due to varying populations examined (sex, race/ethnicity, pre- vs. post-pubertal), baseline serum 25(OH)D levels and strength measures utilized [13, 14, 18, 34, 35]. One study observed a significantly greater grip strength in girls with adequate vitamin D compared to those who were deficient or severely deficient [14], while one small vitamin D supplementation study in post-menarchal females demonstrated no impact of vitamin D supplementation on grip strength [35].

Our results suggest that vitamin D may not modulate the strength/cardiometabolic risk factor relationship even though studies in children and youth have demonstrated a link between serum 25(OH)D and multiple cardiometabolic risk factors [15, 36]. Proposed mechanisms for the impact of vitamin D on cardiometabolic function include the presence of vitamin D receptors on the pancreatic β cells and inflammatory cells [17] and vasculoprotective effects [16]. One vitamin D supplementation study did show promise in improving arterial stiffness among otherwise healthy adolescents with vitamin D deficiency [37]. In the present study, we observed a significant association between vitamin D sufficiency and lower likelihood of borderline/high triglycerides (*p* < 0.05). We also observed a trend toward significance between vitamin D sufficiency and lower odds of overweight/obesity after controlling for covariates (*p* < 0.10). With its known role in muscle contraction [38] and muscle strength in in older adults [39–41], future studies examining the impact of vitamin D supplementation on muscle strength and cardiometabolic risk are warranted.

To our knowledge, this is the first study to examine associations between grip strength and cardiometabolic risk factors, as well as vitamin D status, among a diverse sample of children and adolescents. Due to its cross-sectional design, longitudinal analyses are necessary to draw conclusions about the effect of grip strength on cardiometabolic risk factors. As this study was a secondary analyses of a larger clinical trial, our sample size may be limited to detect relationships between grip strength, blood lipids, and vitamin D status given the relatively low levels of dyslipidemia and vitamin D sufficiency in this population. More specific measures of body composition beyond the use of BMIz would have been useful to better understand the inter-relationships between adiposity, lean mass, strength and dyslipidemia, but we were limited by measurements within the school setting. Furthermore, the measurement of grip strength, although a valid measure of whole body strength, may benefit from additional muscle strength measures, along with other measures such as cardiorespiratory fitness which could contribute to residual confounding. Our analysis, however, was strengthened by the socioeconomic and racial/ethnic diversity of the study population, along with inclusion of a nearly equal percentage of children who were normal weight and overweight/obese. In addition, the age of schoolchildren ranged from 9 to 14 years, allowing both children and adolescents to be included in analyses. Furthermore, the detailed collection of lifestyle factors, sociodemographic characteristics, and anthropometric measures allowed for consideration of various potential confounders. Lastly, grip strength data were robust, as multiple trials were recorded for each hand.

## Conclusion

In conclusion, our findings suggest that greater grip strength is associated with healthier triglyceride and HDL-C concentrations in youth, although these relationships were not independent of BMIz, which implies that it is likely that BMIz is on the causal pathway between these variables. Randomized trials are needed to help delineate which of these explanations holds true. In addition, there was no relationship between grip strength and vitamin D status, suggesting that serum 25(OH)D may not play a role in the relationship between grip strength and cardiometabolic risk factors in this population. While longitudinal analyses are warranted to determine whether grip strength is independently predictive of triglyceride and HDL-C status, muscle-strengthening exercise should nonetheless be considered for enhancing health outcomes in youth.

## Abbreviations

25(OH)D: 25-hydroxyvitamin D
BMI: Body mass index
BMIz: BMI z-score
CI: Confidence interval
DDHS: Daily D Health Study
HDL-C: High-density lipoprotein cholesterol
LDL-C: Low-density lipoprotein cholesterol
OR: Odds ratio
